# Early and severe aortic endograft infection after percutaneous coil embolization of type 2 endoleak: A case report

**DOI:** 10.1016/j.ijscr.2024.110140

**Published:** 2024-08-10

**Authors:** Quentin Balézeaux, Aurélie Leroux, Bruno Krug, Benoit Rondelet, Asmae Belhaj

**Affiliations:** a Department of Cardio-Vascular, Thoracic Surgery and Lung Transplantation, CHU UcL Namur, UcLouvain, Yvoir, Belgium; b Department of Nuclear Medicine, CHU UCL Namur, Godinne Site, Université Catholique de Louvain, Belgium

**Keywords:** Abdominal aortic aneurysm, Coil embolization, Endovascular aneurysm repair, Infection, Homograft, Case report

## Abstract

**Introduction:**

Endovascular aneurysm repair for abdominal aortic aneurysm is superior to open surgery regarding perioperative morbidity and mortality. Complications such as endoleaks represent a significant source of secondary intervention. Vascular graft infection is another serious complication found in 0.2 to 1 % of series. We hereby describe a case of a rapidly progressive aortic infection by Cutibacterium acnes following a percutaneous embolization procedure for a type II endoleak.

**Case presentation:**

A 75-year-old man presented with a fever along with lower back and buttock pain five days after embolization via direct percutaneous puncture for a type II endoleak. White blood cell scintigraphy and blood culture suggested the presence of aortitis, but the patient experienced notable spontaneous improvement in both clinical symptoms and biological markers. The patient underwent CT-angiography which revealed aneurysm rupture requiring urgent open surgery and initiation of antimicrobial therapy. Similarly to blood cultures, per-operative aortic wall tissue samples also revealed presence of Cutibacterium acnes.

**Discussion:**

Aortic endograft infection after embolization is an uncommon complication. The diagnosis is based on a combination of imaging, blood, and nuclear tests. Repeated CT-angiography may be helpful when infection occurs quickly after embolization. Staphylococcus and Streptococci are common pathogens implicated in these infections.

**Conclusions:**

This is a case of an early and severe aortic endograft infection after percutaneous coil embolization of type 2 endoleak. Rupture occurred in less two weeks despite a slow-growing organism infection. The treatment includes endograft removal and antibacterial therapy. Caution is warranted when suspecting aortic endoprosthesis infection, necessitating close follow-up.

## Highlights

• This is a rare case of EVAR infection by Cutibacterium acnes after percutaneous coil embolization.
• EL2 should be managed when the aneurysm has increased by more than 10 mm in diameter.
• VGI often presents with atypical symptoms but patients usually have high white blood cell count, fever, and abdominal pain.
• Repeated CT-angiography and and nuclear imaging may be helpful when infection occurs quickly after embolization.

## 1 Introduction

Endovascular aneurysm repair (EVAR) for abdominal aortic aneurysm is superior to open surgery (OS) regarding perioperative morbidity and mortality [1,2], but long-term survival remains comparable between the two techniques. Endoleaks (EL) represent a significant source of secondary intervention, sometimes occurring several years after the initial procedure [3] and requiring close follow-up when the aneurysm enlarges [4]. Vascular graft infection (VGI) is another serious complication found in 0.2 to 1 % of series [[2], [3], [4], [5]]. We hereby describe a case of an aortitis and rapid EVAR infection following a percutaneous embolization procedure for in our university hospital. This work was reported in line with the SCARE criteria [6] and informed consent was obtained from the patient and his family.

## 2 Case report

**Fig. 1:**
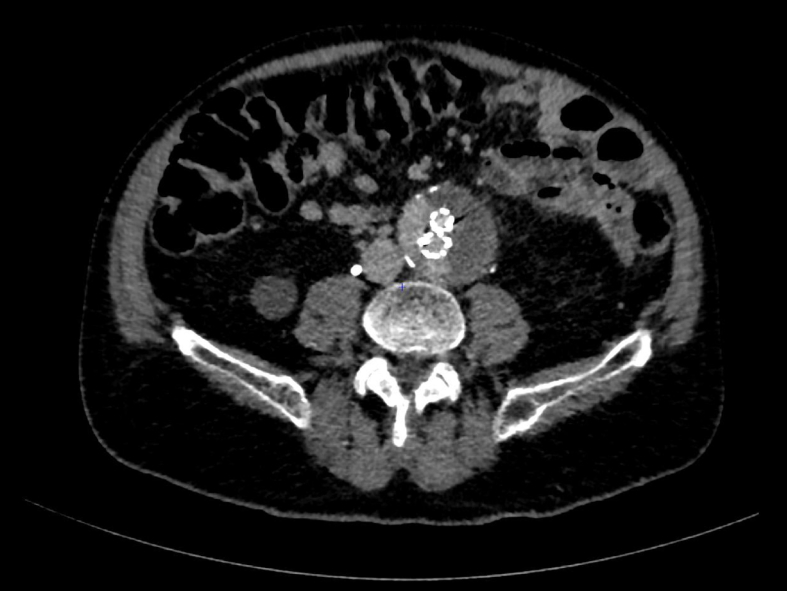
CT scan with contrast, axial view: aneurysmal sac opacification in the right side of the aortic graft.

**Fig. 2:**
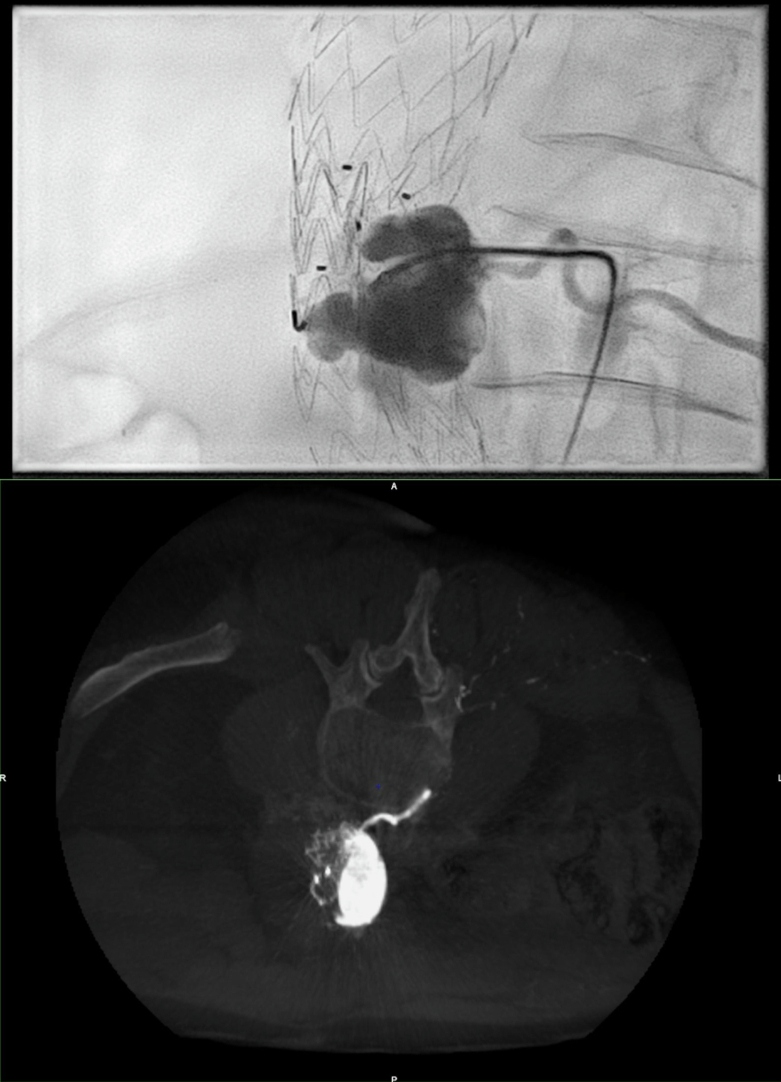
(a) Arteriography and (b) cone bean CT showing embolization of an lombar artery by aneurysmal sac direct puncture.

**Fig. 3:**
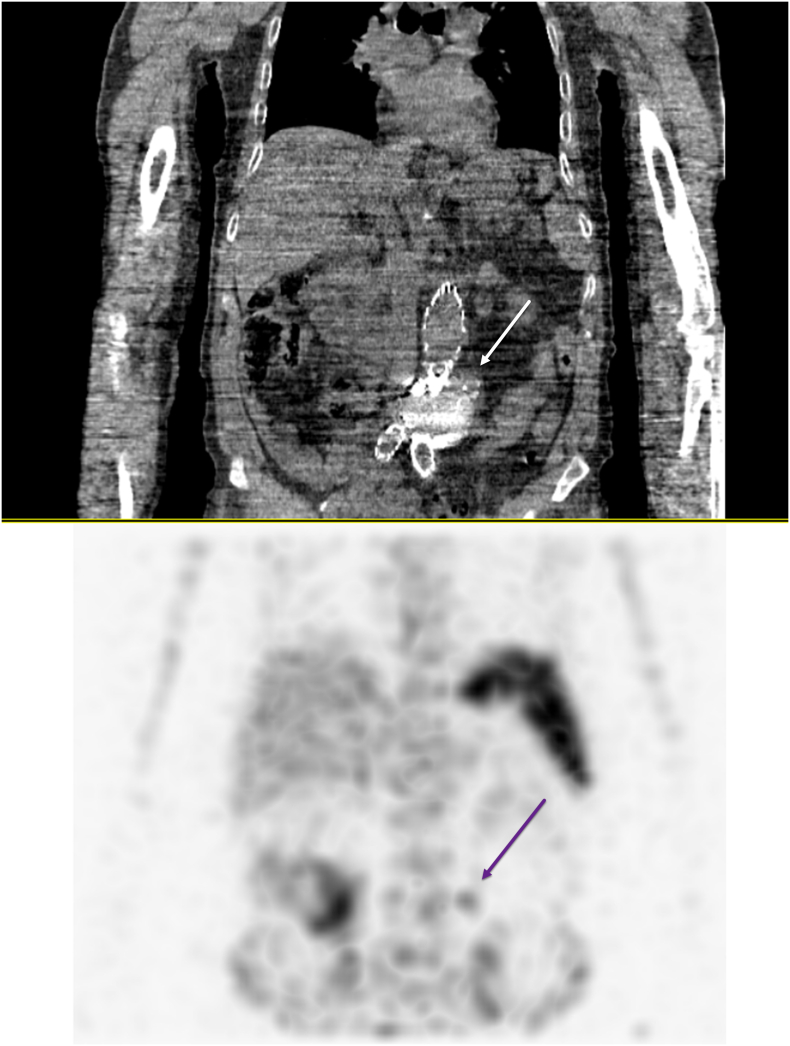
Planar images were taken at 60 min, 4 to 6 h and 24 h post-injection of 285 MBq of ^99m^Tc-HMPAO-labelled white blood cells respectively. The accumulation of white blood cells in two foci was consistent with two septic foci. The SPECT/CT images at 4 to 6 h post-injection confirmed the presence of two foci of white blood cell accumulation in the distal part of the prosthesis.

**Fig. 4:**
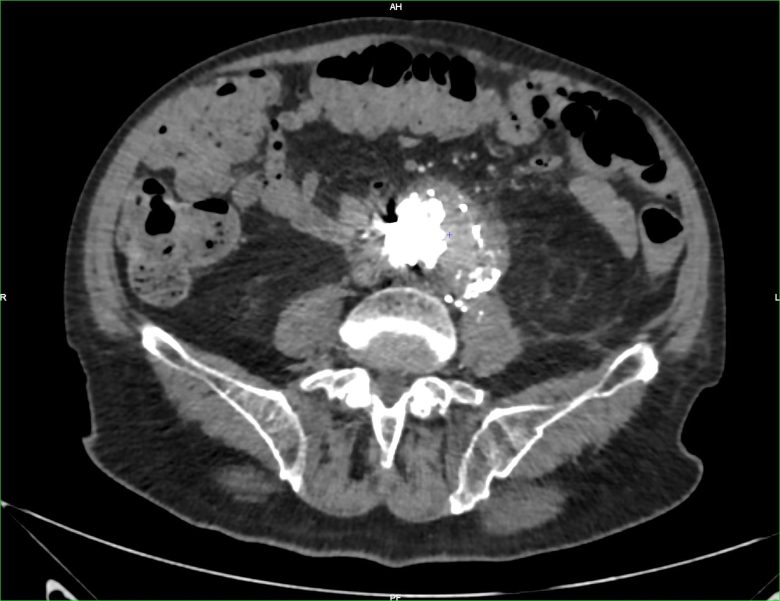
CT scan of the rupture with the modification of the aneurysmal sac on the left side.

**Fig. 5:**
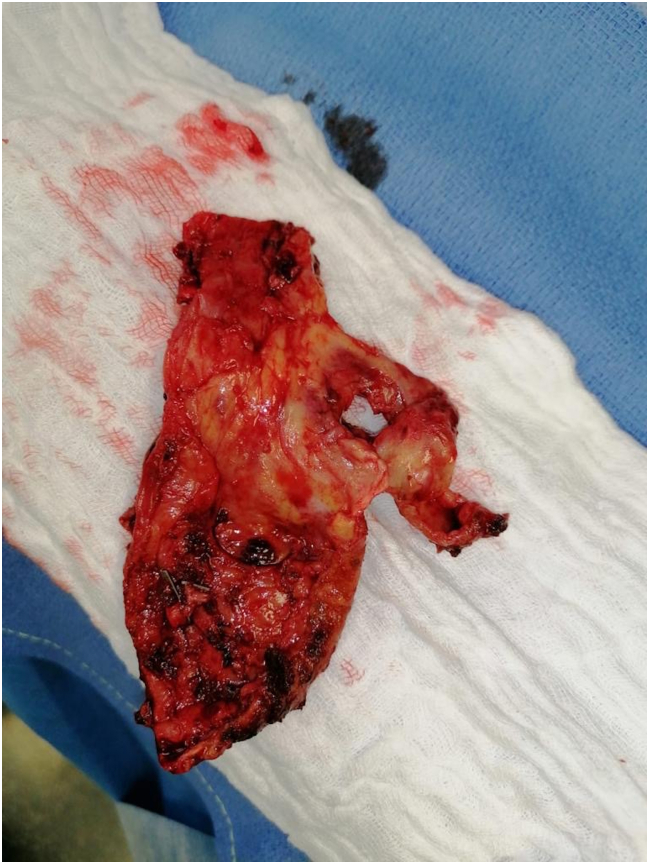
Per-operative photograph of the aorta posterior wall. Arrow showing the rupture site.

A 75-year-old patient underwent an EVAR procedure in 2022. In his medical history, he reports a hemorrhagic stroke, prostatic neoplasia treated with radiotherapy in complete remission, and a partial gastrectomy with vagotomy for gastro-duodenal ulcers. The EVAR procedure and postoperative course were uneventful. A one-year CT-scan follow-up revealed a type 2 endoleak (Fig. 1). The aneurysm size measured 56 mm (2 mm larger than preoperatively). Embolization via direct percutaneous puncture from the left posterolateral approach of the aneurysmal sac was performed (Fig. 2). This procedure involved embolization of a lumbar artery using diluted glue (two vials of Glubran®) with Lipiodol® at a 1:4 ratio. Five months after this procedure, a new CT-scan shows the persistence of the type II EL. A second lumbar artery embolization procedure is performed 17 months after the initial placement of the endograft. The patient was discharged the day after despite one episode of fever attributed to side effect of aneurysmal embolization. Due to the persistence of this fever and lower back and buttock pain, a CT-angiography was performed at day 3, revealing iodinated contrast used for embolization in the aneurysm with some air related to the recent procedure. There was no leakage or morphological changes of the aneurysm. The patient went on to experience daily fever spikes requiring readmission. A white blood cell scintigraphy was undergone and reported high suspicion of early VGI (Fig. 3). Surgical removal of the endograft was recommended. Meanwhile, the patient displayed spontaneous clinical and biological improvement. Surgical intervention was reconsidered, and a new CT-angiography was performed and revealed a modified aspect of the aneurysmal sac, with leakage of embolization material (Fig. 4). Urgent surgical management was decided. Concurrently, a blood culture came back positive for Cutibacterium acnes. The surgical approach was a midline laparotomy. Retroperitoneal exploration revealed no blood extravasation. After removal of the endograft, a localized rupture with purulent infiltration was found in the posterior wall of the aorta (Fig. 5). The subsequent procedure involved excising these infected tissues and performing an aorto-bi-iliac bypass (Intergard Synergy 20/10 prosthesis). Bacteriological tests of the tissues reveal the same Cutibacterium acnes as identified in the blood cultures. The patient received appropriate penicillin-based antibiotic therapy. Ten days after the operation, the postoperative course was satisfactory, including a decrease in white blood cell count and cessation of fever spikes. Unfortunately, the patient developed a spontaneous subdural hematoma with cerebellar compression. Despite prompt decompression by the neurosurgery team, the patient died 22 days after the vascular intervention.

## 3 Discussion

In our knowledge, this is the fourth case of early aortic endograft infection after percutaneous embolization for type 2 endoleak.

VGI is a concerning complication with poor prognosis. Vascular graft infection in the overall population undergoing EVAR was found to be between 0.2 % and 8 %, with an incidence at 25 months of 0.6 % [7]. The average timespan between EVAR surgery and infection is 28.3 months [8]. In our case, the patient presented with infection 17 months after EVAR, with first symptoms appearing within 24 h after the embolization procedure. Postoperative management of EL is a major concerning following EVAR. Type two endoleaks (EL2) are the most frequent.

Under these conditions, it is considered that an EL2 should be managed when the aneurysm has increased by more than 10 mm in diameter compared to the postoperative reference examination (class IIa and level C recommendation from the ESVS [9]). Several endovascular techniques and conversion to conventional surgery are described. The success rate ranges between 84 % and 100 % depending on the techniques. Complication rate is around 4 % for these procedures [10].

Mortality of aortic graft infection is significant, ranging from 20 % to 40 % [11] rising to 63 % without surgery. Several risk factors for infection after EVAR have been identified, including emergency procedures, perioperative complications or additional aortic procedures [7]. Patients usually have high white blood cell count, fever, and abdominal pain. In our case, the patient initially presented with fever. He later went on to present lower abdominal and sacral pain. Surprisingly, the patient never showed signs of severe sepsis and spontaneously improved.

The diagnosis in this case relied on a combination of blood culture identifying the pathogen, CT-angiography, and nuclear imaging. According to the latest ESVS guidelines [12], these tests confirm the presence of inflammation associated with infections. Infection can be diagnosed by white blood cell scintigraphy which detects accumulation of white blood cells over time. Its sensitivity stands at 90 % with a specificity of 88 % [13]. However, accuracy for infection localization is lower compared to PET CT, especially in the aortic region because of the elimination of the tracer by the digestive tract and absorption by the bone marrow. For these reasons, ESVS recommends PET CT for suspected aortic infections (class I and level B [9]). We opted for white blood cell scintigraphy due to its accessibility compared to long PET-CT delays at our institution. The patient's insidious clinical presentation without sepsis or signs of shock also led us to choose this examination over PET-CT. Common pathogens implicated in vascular graft infections include *Staphylococcus aureus*, Streptococci, or *Pseudomonas aeruginosa* [14]. Cutibacterium acnes is less common. This microorganism belongs to the Propionibacterium family, an anaerobic bacterium. This bacterium has been reported in shoulder prosthesis infections, cerebrovascular infections, fibrosis of breast implants, and endocarditis [15]. Cutibacterium acnes is a slow-growing organism with low virulence. In our case, infection and destruction of the aortic wall progressed rapidly. The injection of Lipiodol could have dispersed a significant inoculum directly into the aortic wall. We recommend examining the puncture site to potentially avoid a region with a macroscopically visible follicle. Additionally, it is conceivable that the aortic wall may become poorly vascularized after embolization and the infection spreads quicker in this excluded aneurysmal sac. For these reasons, less virulent pathogens may develop fast. Harry Etienne et al. [16] described a similar case to ours, with an EVAR infection by Cutibacterium acnes occurring 7 days after percutaneous embolization for EL2. They observed peri-aortic infiltration on day 9, followed by liquid collection on day 16. Inflammation was confirmed using PET-CT. In our case, the follow-up CT-angiography on day 10 confirmed infectious progression, despite our patient's favorable evolution. This case underscores the importance of repeated imaging in suspected VGI following embolization procedures. In another case report by Florian Dick et al. [17], two cases of aortitis following EVAR endoleak embolization were described. For the first case, the patient experienced abdominal pain, nausea, vomiting, and increased white blood cell count two days after the intervention. A CT-angiography performed at day 11 revealed an increase in the size of the aneurysmal sac. They undertook emergent surgical intervention and blood cultures tested positive for Staphylococcus and Streptococcus. In their second case, a patient was readmitted 21 days after percutaneous embolization. The patient complained of pain in the iliac fossa and right buttock. A CT-angiography showed aortic wall thickening. Surgical specimens revealed the presence of Cutibacterium acnes in the operative sites. The clinical course of these patients is similar to ours, with signs of infection identified on the CT-angiography between 10- and 20-days post-procedure. In our hospital, percutaneous trans-lumbar embolization is done by the interventional radiology team. Embolization is performed under local anaesthesia, guided by CT scan.

The diagnosis of endograft infection necessitates conversion to open surgery, with removal of the infected graft and reconstruction [18]. Extra-anatomic reconstruction could be accomplished with axillobifemoral bypass. This option avoids reintroducing foreign tissue into the infected area. In-situ reconstruction is also well-described. It can be performed using autologous superficial femoral veins. Cryopreserved allografts have a low reinfection rate, but tissue degradation over time necessitates additional intervention, with rates up to 55 % at 5 years [19]. New prosthetic material must be protected by rifampicin or silver-coated materials. Management of endograft infections require a multidisciplinary approach, and close collaboration with infectious disease specialists for targeted antibiotic therapy that may be continued for several weeks. We are mindful that our experience cannot be generalized. Larger studies need to be carried out on this complication to identify the best treatment.

## 4 Conclusion

We report a rare case of early infection of an aortic endoprosthesis following embolization of an EL. The identified microorganism is known to be low-virulent. White blood cell scintigraphy and repeat CT-angiography played crucial roles in establishing a definitive diagnosis. Despite favorable clinical and biological evolution, the patient developed a severe infection at the surgical intervention site. Therefore, caution is warranted when suspecting aortic endoprosthesis infection, necessitating close follow-up. More large series are required to have solid data and conclusions. This work was made possible with the help of the Fondation Mont Godinne.

## Declaration of competing interest

The authors declare that they have no known competing financial interests or personal relationships that could have appeared to influence the work reported in this paper.
